# An empirical assessment of electricity consumption and environmental degradation in the presence of economic complexities

**DOI:** 10.1007/s11356-022-21099-9

**Published:** 2022-06-11

**Authors:** Elma Satrovic, Festus Fatai Adedoyin

**Affiliations:** 1 Hasan Kalyoncu University, Gaziantep, Turkey; 2 Department of Computing and Informatics, Bournemouth University, Bournemouth, UK

**Keywords:** Ecological footprint, Economic complexities, Electricity consumption, Environmental degradation, O13, P48, Q43

## Abstract

To a large extent, the theories and concepts behind the effect of ecological footprint have been the paramount concern of the recent literature. Since the rising and falling of environmental degradation have been a continuous issue since the first phase of development, determinants such as economic complexity may play a critical role in achieving long-term sustainable development in the framework of environmental Kuznets curve (EKC) paradigm. Therefore, this research expands on the notion of an EKC paradigm for the world’s top ten most complex economies by considering four variables, such as real GDP per capita, electricity consumption, trade openness, and a new putative factor of environmental obstacle, the economic complexity index (ECI). This is one of the first studies to look at the impact of ECI on the ecological footprint of a specific sample from 1998 to 2017. The findings demonstrate a continuous inverted U-shaped link between real GDP per capita, the square of real GDP per capita, and ecological footprint. The EKC hypothesis is found to be valid in the long term in the examined complex economies. The findings of the panel autoregressive distributed lag (ARDL) of the pooled mean group (PMG) and fully modified ordinary least squares (FMOLS) estimations demonstrate that in the long term, electric power usage contributed to the carbon footprints. Furthermore, the economic complexity index and trade openness increase environmental performance over time. To determine if there is causation between the variables, we employ the panel vector error correction model (VECM) framework. Particularly, the results show unidirectional causality running from electric power consumption to ecological footprint and bidirectional causal relationship between (1) economic growth and ecological footprint; (2) square of economic growth and ecological footprint; (3) economic complexity index and ecological footprint; and (4) trade openness and ecological footprint.

## Introduction

Climate change is the greatest and most urgent global challenge with long-term implications that can influence a transition toward sustainability of all countries. As a component of climate change, global warming is an unavoidable ecological hindrance induced by human activities that raise the quantities of greenhouse gases in the atmosphere. The environmental impact of economic growth includes increased energy consumption and higher levels of pollution as posited by Bekun (2022) and Adedoyin et al. (2021a). In other words, the burning of fossil fuels is one of the major sources of human-generated emissions. The production and use of electricity as a primary source of energy have put significant strain on the environment (Ahmad and Wu 2022). Anthropogenic greenhouse gas emissions affect air, water, and soil quality at both local and global levels (Alves and Uturbey 2010; Satrovic and Dag 2019; Isik et al. 2020; Yildiz 2021, 2022). Electricity powers industry, education, communications, healthcare, entertainment and is the heart of modern economies. As such, many regions especially developing have significant growth in electric power consumption. According to the International Energy Agency (IEA), worldwide annual electricity demand will rise by 2.1% from 2018 to 2040, raising electricity’s proportion of gross electricity consumption from 19% in 2018 to 24% in 2040. Many academics have lately examined the influence of electricity usage on productivity expansion to the causal relationship between the two factors mentioned on ecological restrictions, and this arguing point is commonly referred to as the environmental Kuznets curve (EKC) theory (Neagu 2019; Verbič et al. 2021; Satrovic et al. 2021; Adedoyin et al. 2021b; Satrovic and Abul 2022).

Grossman and Krueger’s (1991, 1995) investigations provided a solid foundation for this concept. The EKC theory is a pictorial depiction of an inverse U-shaped that implies that ecological hindrance grows in the initial phases of development until it approaches a saturation point where it declines with economic progress (Mealy and Teytelboym 2020; Ozden and Bese 2021; Can et al. 2021; Abul and Satrovic 2022). Given that CO2 is acknowledged to be the most prominent greenhouse effect gas (GHG), it is often employed as an indicator of environmental deterioration (OECD 2004). While the statistics on carbon dioxide emissions only report tons of emissions annually, ecological footprints are usually reported in comparison to what the planet can renew (Yilanci and Pata 2020). In this work, we employ ecological footprint as a more thorough index of environmental deterioration. Rees and Wackernagel (1996) created a sophisticated technique for determining human carrying capacity. This indicator addresses the issue of how much of the Earth’s surface is appropriated to support the “load” imposed by a reference population, regardless of its reliance on commerce or level of technological development. Besides air pollution, this indicator also accounts for water and soil pollution. The indicator called ecological footprint (EF) is the only metric that tracks how much nature we have and how much nature we use and is expressed in global hectares. The ecological footprint is a measure that considers human demand for world biodiversity while also reflecting complicated ecological barriers.

Most of the research on the EKC hypothesis proxied economic growth for real GDP per capita (Can and Gozgor 2017; Zhang et al. 2021; Laverde-Rojas et al. 2021; Boleti et al. 2021; Adedoyin et al. 2022). However, the environmental constraint is a problem that extends beyond production numbers because real GDP per capita does not completely convey an economy’s structural changes. Arab nations (for example, Saudi Arabia, Qatar, and the United Arab Emirates) have a comparatively low complexity of export base but a relatively high real GDP per capita in contrast to the rest of the globe. Hidalgo and Hausmann (2009) developed the notion of economic complexity and offered the Economic Complexity Index (ECI) as a follow-up comprehensive tool to better quantify and comprehend the structure of economy. The ECI assesses the productive knowledge exhibited in the examined state’s financial operations. In other words, it looks to measure the productive capabilities of the inspected economic system. Countries with higher economic complexity, like Japan or Switzerland, export many sophisticated and diverse products and report high ECIs. Countries with lower economic complexity (low ECI), like Nigeria or Angola, have little diversity and export less sophisticated products. At this stage, economic structural transformation processes should be assumed to lessen environmental obstacles since this process increases levels of capital intensity and introduces new technology. Unarguably, this process transforms economies from “energy intensive” to “technology intensive” (Can and Gozgor 2017; Incekara 2019). Economic complexity index, as a determinant of ecological impediment, suggests that countries with lower complexity generally produce agricultural products and cause limited environmental degradation, whereas more complex countries cause excessive environmental degradation. However, the topmost complex economies around the globe introduced better technology and behave more environmentally friendly (Yilanci and Pata 2020). Clean production technologies reduce the environmental degradation of the topmost complex economies. In recent times, the environmental impacts of trade openness are an issue of hot debate and growing importance in trade policy. Tachie et al. (2020) summarize the three main effects—the scale, the composition, and the technique effects—explaining the trade openness-environment nexus. As a result of high energy consumption and production, the scale effect shows a positive association between trade openness and environmental deterioration. The composition impact is related to the manufacturing structure, whereas the technique effect introduces cleaner technologies and reduces environmental deterioration.

The EKC theory has been thoroughly examined for many groupings of nations. However, to the authors’ knowledge, scientific findings on the consequences of economic complexity on the environment are extremely sparse. The propositions of Chu (2020), who suggests that evolution in economy’s knowledge reduces energy consumption and introduces environmentally friendly production technologies, motivated to investigate the influence of economic complexity in the world’s top ten most sophisticated economies. Economic expansion, energy usage, the economic complexity index, and trade openness can all have an impact on environmental constraints for the reasons above. Therefore, the major objective of our article in this context is to evaluate the validity of the EKC hypothesis in the top ten most complex economies, while investigating the combined impact of real GDP per capita, electricity consumption, trade openness, and economic complexity index on ecological footprint.

Despite that the roles of real GDP per capita, electricity consumption, trade openness, and economic complexity index in explaining environmental obstacle have been the paramount concern of the past literature, some important aspects remain unaddressed. In four ways, our study addresses these aspects to add to the available knowledge pools. *Firstly*, to avoid estimate bias, we account for complexity in the electricity usage degradation link. *Secondly*, we increase the estimates even more by modeling environmental deterioration using the ecological footprint, which is a more comprehensive indicator of environmental degradation than carbon emissions. This aspect is critical since ecological footprint is a measure that considers human demand for world biodiversity while also reflecting complicated ecological barriers. *Thirdly*, to produce more robust conclusions, the long-run elasticity of economic complexity and other factors are calculated using different panel data econometric methodologies. *Finally*, to the best of our knowledge, this is the first attempt to examine the influence of economic complexity on environmental footprint among the top ten most complex economies. Economic structural transformation processes should be assumed to lessen environmental obstacles since this process increases levels of capital intensity and introduces new technology. Furthermore, concentrating on nations with comparable economic complexity improves the consistency and efficiency of the estimations.

The following research questions are raised in response to the ongoing conversation. Is there a validation of EKC hypothesis correct for an ecological footprint in the world’s top ten most complex economies? Is there a long-term link between growth, economic complexity, electricity use, trade openness, and environmental footprint?

The remainder of the research is organized as follows. The next part provides a thorough assessment of the literature on the relationship between investigated variables. The data and econometric methods utilized to reach the study’s goal are presented in the next section. The “Results and discussion” section summarizes the findings and examines the study’s different ramifications. Finally, “Conclusion and policy recommendation” provides a conclusion as well as policy implications for the selected sample of nations.

## Literature review

To a large extent, the theories and concepts behind the effect of ecological footprint and other greenhouse gas emissions have been discussed in the introductory section. It was also discussed that the advancement in the economic growth of countries leads to dangerous hazards being emitted into the environment. Four variables, such as real GDP per capita, electric power consumption, trade openness, and economic complexity index were identified and referenced to be the determinant of ecological footprint, especially in the top complex economics. However, in empirical reviews relationships shall be the focus of this section, as with other similar research in this area such as Gyamfi et al. (2021a, b, c). The word ecological footprint as well as carbon emission, environmental degradation would be used interchangeably because they are greenhouse gas emissions and, of course, they all both have toxic effects on the quality of the environments. Thus, this section will be divided into two subsections which are environmental degradation and economic complexity: and environmental degradation, economic growth, electricity consumption, and other variables.

### Environmental degradation and economic complexity

The rising and falling of environmental degradation have been a continuous issue since the first phase of development, where agricultural-based products are the specialties of most countries (Doğan et al. 2019; Gyamfi et al. 2020) before gradual movement into the industrial-based product (Dinda 2004). Environmental awareness is quite low at this level, and the manufacturing of polluting items is expanding. Consequently, Gozgor and Can (2016) avowed that more energies, which is bad for the environment, are consumed by the countries. However, with a certain amount of increasing economic growth, the countries shift increasingly toward technology-oriented industry, and thus the society’s ecological awareness grows, and the country ceases the manufacture of polluting items. Therefore, carbon emission or ecological footprint is reduced through the allocation of countries’ production factors toward technology-intensive techniques (Apergis et al. 2018).

Furthermore, as technology advances, the procedures employed in manufacturing will be more sophisticated and greener. Less energy will be utilized in manufacturing due to technology-intensive creative production procedures, resulting in lower pollutant emissions (Shahbaz et al. 2018; Yin et al. 2015). Put differently, among the most critical elements that countries used as yardsticks to reduce emission are the technology utilized in their manufacturing processes (Lorente et al. 2018). These last sentences by Lorente et al. (2018) reflect that the production capacity of some countries is dependent on the variables factor into the production process. This is following what Doğan et al. (2019) posited in his research, that is, the strongest information on a country’s technological level and production factors are gained from the items that country produces, meaning that the production of more advanced goods depicted that the elements of the production are acceptable.

The level of the technical production of sophisticated goods and intensive-based production structure is what is termed economic complexity, which according to Can and Gozgor (2017) provides vital information on a country’s economic structure and its level of innovation (Doğan et al. 2019). As Hidalgo (2009) posited, complexity reflects a country’s strengths and competencies in terms of goods and industrial products, and the high rating of economic complexity reflects the sophistication of the nations’ commodities. When talking of ECI, it is the measure of countries’ technology-based capacities in the construction of diverse goods and services, the extent of scale, structure, and innovation changes of a country, and amount of export basket of the country. For instance, Japan and Switzerland with the highest ECI produce more technological-based products and have large export baskets. Undeniably from the above pieces of literature, ECI contributes significantly to determining the level of real GDP per capita (Hausmann et al. 2007; Strojkoski and Kocarev 2017).

Since the need to foster economic growth has the consequence of degrading the environmental quality through industrial production and advancement in technology level of countries as stated by Yin et al. (2015), and since economic complexity is tantamount to the country has sophisticated manufacturing processes through technology-intensive techniques, then it is expected to have its effects on the quality performance of the environment. As a result, various researchers, although few, have taken the ground of exploring whether ECI has positive or inverse effects on the quality of the environments. The first-ever research, which used dynamic ordinary least square (DOL), to examine the impact of ECI on the environment is the study of Can and Gozgor (2017) where France was used as a case study. The result of the study affirmed the existence of the EKC hypothesis and the reduction of environmental emission due to the ECI of the country. The result contradicts what Neagu and Teodoru (2019) observed European Union (EU) was used as a case study, that is, Neagu and Teodoru (2019) revealed that ECI measured the increase in environmental emission in the EU countries. In the research of Shahzad et al. (2021), a range of econometrics methods was applied to assess the effects of ECI on ecological footprint using US quarterly data from 1965 to 2017; the outcome showed that ecological footprint and ECI are causally related and that ECI fosters ecological footprint in the USA. This is similar to the result of Ulucak and Lin (2017) who also opined that change in climate policies influenced ecological footprint in the USA.

### Environmental degradation, economic growth, energy use, and other variables

Although there is a rarity of papers on ECI and ecological footprint, nonetheless, there have been several studies on what influences the ecological footprint. Danish and Wang (2019) investigate the factors that determine the ecological footprint of NEXT-11 nations. The variables considered were urban growth and energy consumption. The result revealed that ecological footprint is enhanced by urban growth and extensive consumption of energy. The research advised non-retardation of economic growth of the NEXT-11 countries with the release of less or no harmful substances into the environment. In the same vein, the ecological footprint of 16 EU countries was investigated by Alola et al. (2019), but with trade policy in place of urban growth. The result revealed significant damage to the environment from the economic growth and consumption of nonrenewable energy, although utilization of renewable energy was shown to alleviate the ecological footprint in the regions.

Ozcan et al. (2019) investigated the role of climate change regulations in low-, middle-, and high-income countries. The empirical findings revealed that increases in the ecological footprints of high-income and middle-income countries are temporary and may rebound to their previous pattern quickly after being disrupted by energy market, economic sector, or environmental shocks. More specifically, the researchers concluded that policymakers should concentrate on long-term green regulation instead of setting superfluous objectives in response to disruptions in the carbon footprint. In other research, Zafar et al. (2019) uses the USA as a case analysis to explore the impact of natural resources, foreign investments, human capital, and economic growth on the ecological footprint. Except for economic development, the factors analyzed are favorable to minimizing the ecological footprint, according to the empirical results of the autoregressive distributive lag (ARDL) model. From a policy aspect, the study suggested that the USA undertake reforms to increase nature’s bio-productivity for sustainable and harmless expansion, such as rehabilitation and soil management measures.

Sharif et al. (2020), using quantile ARDL, explored the influence of the tourist industry and industrialization on environmental stewardship and the viability of the EKC hypothesis over quarterly data in China. The study revealed that economic expansion in China worsens environmental deterioration, and that the tourist industry causes environmental externalities in China, proving the validity of the EKC concept. Rahman (2020), in his research, discovered that electricity usage worsens the environment for the panel of G7 nations as well as the UK as a country. On the other side, electricity consumption had a favorable impact on the carbon emission when Jalil and Feridun (2011) studied China as a reference country and Hossain (2011) studied global industrialized economies.

Also like these results, as studied by Wang et al. (2018), are the positive effects of electricity consumption on environmental emission. The emission-economic growth nexus study of Chakamera and Alagidede (2018) also noted that energy consumption is indeed an important variable that policy should watch for in maintaining a sustainable environment. The more conflicting result is observed by Nathaniel and Khan (2020) when they investigated the impacts of electricity usage—both renewable and nonrenewable energies, real GDP per capita, and urbanization on ASEAN nations’ ecological footprints. The panel cointegration and regression results demonstrated that both economic growth and non-renewable energy had a considerable negative impact on the environment in ASEAN economies. According to the findings, the ASEAN region’s economy is rising at the price of climate change and sustainable production.

## Data and methodology

### Data

This study used an annual data, covering the period of 1998–2017 for the 10 most complex countries around the globe. The choice of the study period is dictated by the availability. The selected samples according to their 2018 economic complexity index (Growth Lab at Harvard University 2019) include Japan (2.43), Switzerland (2.17), South Korea (2.11), Germany (2.09), Singapore (1.85), Austria (1.81), Czech Republic (1.80), Sweden (1.70), Hungary (1.66), and Slovenia (1.62). The unavailability of data for economic complexity beyond 2018 dictated the selection of countries sample. As we try to evaluate the linkage amid economic complexity and ecological impediments, we make use of traditional variables to certify the validity of the hypothesis of an EKC. However, as a novelty, we introduce ecological footprint and ECI in the EKC model to test the association between the selected macroeconomic variables and ecological impediments. The summary of the data set is signified in Table 1.

**Table 1:** Definition and data source

Indicator name	Code; measurement unit	Source
Ecological footprint	ECFP; gha per person	Global Footprint Network (2021)
Economic complexity index	ECI; ranking	Simoes and Hidalgo (2021)
Real GDP per capita	GREC; constant 2010 US$	World Bank (2021)
Electric power consumption	PEC; kWh per capita	World Bank (2021); IEA (2021)
Trade	OTT; % of GDP	World Bank (2021)

Source: authors’ compilation from Global Footprint Network (2021), Simoes and Hidalgo (2021), World Bank (2021), and IEA (2021)

The per capita ecological footprint of the inspected countries is sourced from the Global Footprint Network (2021) and illustrated as the function of economic complexity index published by the Observatory of Economic Complexity (2021), real GDP per capita measured in constant 2010 USD, trade openness measured as % of GDP sourced from the World Bank (2021), and electric power consumption measured in kWh per capita sourced from the World Bank (2021) and International Energy Agency (2021). The primary attributes of the inspected variables are given in Table 2.

**Table 2:** Descriptive statistics and correlation matrix

Stat./Var.	ECFP	GREC	PEC	ECI	OTT
Austria					
Mean	6.02	45,570.74	7943.43	1.56	94.97
SD	0.31	2914.60	560.42	0.05	9.27
Maximum	6.55	49,078.90	8548.88	1.61	105.15
Minimum	5.56	39,443.90	6706.71	1.45	76.93
Switzerland					
Mean	5.30	74,585.61	7919.16	1.87	107.33
SD	0.40	4704.72	305.41	0.09	13.56
Maximum	5.86	80,964.60	8360.58	2.05	130.89
Minimum	4.47	66,460.80	7468.88	1.75	87.74
Czech Republic					
Mean	5.88	18,650.13	6189.76	1.47	122.73
SD	0.47	2736.10	315.84	0.20	24.28
Maximum	7.22	23,144.40	6557.05	1.69	157.58
Minimum	5.32	14,066.90	5493.85	1.10	84.42
Germany					
Mean	5.20	41,233.66	6998.97	1.91	73.91
SD	0.26	3273.66	249.76	0.07	11.97
Maximum	5.69	46,907.80	7281.27	2.05	87.41
Minimum	4.70	36,285.90	6479.49	1.78	51.59
Hungary					
Mean	3.66	12,933.17	3778.86	1.20	145.05
SD	0.41	1705.08	290.84	0.28	21.05
Maximum	4.63	15,912.00	4263.41	1.50	168.24
Minimum	2.93	9679.21	3215.68	0.71	107.43
Japan					
Mean	4.89	44,421.52	8175.15	2.13	27.77
SD	0.27	2122.93	274.38	0.14	6.31
Maximum	5.28	48,510.60	8710.03	2.31	37.55
Minimum	4.44	41,098.00	7792.09	1.82	18.35
South Korea					
Mean	5.49	20,872.59	8375.86	1.37	78.07
SD	0.51	4364.83	2020.02	0.40	15.14
Maximum	6.18	27,492.60	10,754.60	1.86	105.57
Minimum	3.86	12,877.40	4436.17	0.68	58.35
Singapore					
Mean	6.73	43,358.38	8346.10	1.42	366.02
SD	0.62	8651.32	642.29	0.28	37.11
Maximum	8.26	57,527.60	9248.49	1.76	437.33
Minimum	5.85	30,116.50	6965.75	0.80	303.32
Slovenia					
Mean	5.01	22,147.64	6585.40	1.34	124.95
SD	0.41	2557.30	504.12	0.16	19.93
Maximum	5.84	25,754.70	7230.69	1.55	157.28
Minimum	4.49	17041.50	5574.71	1.05	92.54
Sweden					
Mean	6.33	50,803.04	14,766.89	1.75	82.38
SD	0.63	4716.58	869.46	0.05	4.80
Maximum	8.38	57,467.30	16,021.00	1.85	92.56
Minimum	5.28	41,247.60	13,457.50	1.68	74.34
Correlation matrix	ECFP	GREC	PEC	ECI	OTT
ECFP	1				
GREC	0.323^***^	1			
PEC	0.595^***^	0.501^***^	1		
ECI	0.098	0.647^***^	0.427^***^	1	
OTT	0.351^***^	-0.019	-0.089	-0.319^***^	1

Source: authors’ compilation from Global Footprint Network (2021), Simoes and Hidalgo (2021), World Bank (2021), IEA (2021)

***, **, and * represent statistical significance at the 1%, 5%, and 10%, respectively

As can be seen in Table 2, ecological footprint has an average of 5.45 with a standard deviation of 0.93. Of the ten economies inspected, Singapore has the highest mean ecological footprint, followed by Sweden, Austria, and the Czech Republic. The lowest average ecological footprint is reported for Hungary. Table 2 portrays that, regarding the selected sample of the top 10 most complex economies, real GDP per capita is reported to be averagely 37,457.65 (2010 US$), with a minimum value reported for Hungary and the maximum value of reported for Sweden. Meanwhile, an inspection of the findings outlines a high average level of economic complexity index in Japan and Germany. ECI is evidenced to be averagely 1.60, with a maximum value of 2.31 reported for Japan and a minimum value of 0.68 reported for South Korea. Considering the mean values, electric power consumption is one of the predictor variables incorporated in the ecological footprint function, which had the largest average value of 14,766.89 in Sweden with a standard deviation of 869.45. For group summary statistics, the electric power consumption shows an average value of 7907.96 with a dispersion value of 2764.21. Singapore leads the way in terms of average trade (as a percentage of GDP), while Japan has the lowest. The average value of group summary statistics (OTT) was 122.32, with a standard deviation of 89.14.

Table 2 further depicts the correlations that exist amid ecological footprint, real GDP per capita, electric power consumption, economic complexity index, and trade openness. The outcome outlines a positive link between ecological footprint and real GDP per capita. The outcomes also indicated that electric power consumption is correlated positively with the ecological footprint. In addition, trade openness is positively correlated with the ecological footprint. While other variables are positively linked with ecological footprint, the economic complexity index exhibits correlation that is not statistically significant. Real GDP showed a positive connection between electric power consumption and ECI. Electric power consumption as well exhibited a positive correlation with ECI while ECI showed a negative correlation with trade openness. Notably, electric power consumption reveals the highest correlation, which shows that electric power consumption has a substantial impact on ecological footprint among the sample countries.

### Theoretical framework and model specification

Various empirical evidence authenticated in the literature has clarified the economic growth-carbon emission nexus (Ahmad et al. 2021; Satrovic et al. 2021; Khan et al. 2022; Abul and Satrovic 2022). While several others have been conducted on economic complexity-carbon emission nexus (Can and Gozgor 2017; Doğan et al. 2019; Chu 2020; Laverde-Rojas et al. 2021), while some have focused on trade openness-carbon emissions link (Tachie et al. 2020; Satrovic 2019a). However, this study is an attempt to introduce economic complexity as an explanatory variable of ecological footprint together with economic growth, electricity use, and trade openness as predictors of ecological impediments. Besides, our study is paramount from the perspective that it uses ecological footprint instead of carbon dioxide emission to probe into novelties contrasting with the bulk of the other studies on the EKC hypothesis. Our study analyzes the authenticity of the EKC hypothesis in the ten most complex economies around the globe such that underlying model can be formalized as (Eq. 1)

1 $$ECFP=f\left( GREC,{GREC}^2, PEC, ECI, OTT\right)$$

where GREC and GREC^2^ abbreviate for the economic growth and its squared term, respectively.

Furthermore, all variables are represented as natural logarithms to guarantee that the variance remains consistent throughout all series. Especially, the coefficients with the log-linear variables will be interpreted as elasticities. Hence, the augmented ecological footprint function in a panel specification is formalized as (Eq. 2)

2 $$L{(ECFP)}_{it}={a}_0+{\beta}_1L{(GREC)}_{it}+{\beta}_2L{(GREC)}_{it}^2+{\beta}_3L{(PEC)}_{it}{\beta}_4L{(ECI)}_{it}+{\beta}_5{(OTT)}_{it}+{\varepsilon}_{it}$$

where *i* signals the top 10 most complex economies around the globe and *t* abbreviates time period; *β*_1_⋯*β*_5_ stand for ecological footprint elasticities concerning economic growth, economic growth squared, electricity consumption, economic complexity index, and trade openness; *a*_0_ is the intercept; *ε*_*it*_ is the error term; and *L* stands for natural logarithm. Several past studies (Nathaniel and Khan 2020; Neagu 2020; Shahzad et al. 2021) measured environmental obstacle by ecological footprint to address the issue of how much of the Earth’s surface is appropriated to support the “load” imposed by a reference population, regardless of its reliance on commerce or level of technological development. Besides air pollution, this indicator also accounts for water and soil pollution. This indicator is the only metric that tracks how much nature we have and how much nature we use and is expressed in global hectares. The ecological footprint is a measure that considers human demand for world biodiversity while also reflecting complicated ecological barriers. Ecological footprint has been acknowledged to be driven by various macroeconomic factors. The advancement in the economic growth of countries leads to dangerous hazards being emitted into the environment. The existence of EKC phenomenon will be validated if ecological hindrance grows in the initial phases of development until it approaches a saturation point where it declines with economic progress (*β*_1_ > 0; *β*_2_ <0). Most previous works (Laverde-Rojas et al. 2021; Satrovic et al. 2021; Khan et al. 2022) considered real GDP per capita to investigate the validity of EKC hypothesis. Environmental impact of economic growth can be observed from the perspective that economic progress includes increased energy consumption leading into more anthropogenic emissions. Also, *β*_3_ is speculated to be positive referring to electric power consumption to explain the adverse environmental impact. The impact of electricity consumption on ecological footprint is captured by PEC lending credence to (Satrovic 2019b; Murshed 2020; Fahad and Wang 2020). The burning of fossil fuels is one of the major sources of human-generated emissions. The production and use of electricity as a primary source of energy have put significant strain on the environment (Ahmad and Wu 2022). Electricity powers industry, education, communications, healthcare, and entertainment and is the heart of modern economies. As such, it plays a critical role in maintaining a sustainable environment. Furthermore, *β*_4_ as authenticated by Chu (2020) and Doğan et al. (2019) is expected to have a negative sign showing that complex countries may accumulate environmental knowledge and utilize environmentally friendly technology. With a certain amount of increasing economic growth, the countries shift increasingly toward technology-oriented industry, and thus the society’s ecological awareness grows, and the country ceases the manufacture of polluting items. Furthermore, as technology advances, the procedures employed in manufacturing will be more sophisticated and greener. Less energy will be utilized in manufacturing due to technology-intensive creative production procedures, resulting in lower pollutant emissions. Finally, the elasticity of trade (*β*_5_) is expected to be negative. A negative relationship may suggest that trade liberalization increases the demand for environmentally friendly goods and services. In addition, trade policies have helped complex countries to control pollution. Some scholars (Tachie et al. 2020; Satrovic 2019a) used OTT to determine its influence on environmental obstacles.

### Estimation methods

The estimation strategy first adopted the cross-sectional dependence (CD) test. In testing for relationship between variables in the long run, one concern is to figure out whether increased economic integration caused a cross-sectional dependence of error terms. Ignoring the issues of cross-sectional connectedness may cause potential bias and provide unreliable outcomes. Hence, we used CD test of Pesaran (2004) to look for the cross-sectional dependence defined by

$$CD=2T{\left(N-1\right)}_i=1N-{1}_j=i+1{N}_{\rho ij}$$

Pesaran (2004) CD test is used to address large *N* bias of the Lagrange multiplier (LM) test.

Concerning the second stage of the analytical procedure, it is admired to have the variables of the same order of integration. Thus, in the second stage of the analysis, we employed the recently developed cross-sectional augmented panel unit root test (CIPS) to investigate the integration properties. The CIPS is a second-generation unit root test developed by Pesaran (2007) that considers the potential CD in panel data. Herein, the great advantage of CIPS test in comparison with first-generation unit root tests is the ability to solve the problem of inefficiency in the estimation.

The regression of the cross-section augmented DF (CADF) test is estimated to obtain CIPS test statistics as follows:

$$CIPS=\frac{1}{N}\sum_{i=1}^N{CADF}_i$$

Much like the CD, it is pertinent to evaluate the long-run linkage amid the ecological footprint, economic growth, economic growth squared, electric power consumption, economic complexity index, and trade openness. In other words, we are ascertaining the cointegration link among the inspected variables by using the Westerlund (2007) cointegration test and Kao (1999) residual cointegration tests. Westerlund (2007) developed four new cointegration tests that are normally distributed and address cross-sectional dependence as well as the short-term dynamics in *i*. The four tests are presented as

$${G}_{\tau }=\frac{1}{N}\sum_{i=1}^N\frac{{\hat{a}}_i}{SE\left({\hat{a}}_i\right)},{G}_{\alpha }=\frac{1}{N}\sum_{i=1}^N\frac{T{\hat{a}}_i}{{\hat{a}}_i(1)},{P}_{\tau }=\frac{{\hat{a}}_i}{SE\left({\hat{a}}_i\right)},{P}_{\alpha }=T\hat{a}$$

where $SE\left({\hat{a}}_i\right)$ captures the standard error of ${\hat{a}}_i$

To evaluate the authenticity of U-shaped relationship, our study uses the panel autoregressive distributed lag (ARDL) of the pooled mean group (PMG) and fully modified ordinary least square (FMOLS) for the panel cointegration regression. Pedroni (2000) proposed the FMOLS technique that approves the validity of per capita real GDP and other predictor variables in explaining the ecological footprint in the top 10 most complex countries around the globe. The panel FMOLS estimator is explained in detail in Pedroni (2000) and can be formalized as (Eq. 3)

3 $${\beta}_{FMOLS}={\left[\sum_{i=1}^N\sum_{t=1}^T{X}_{it}{X}_{it}^i\right]}^{-1}\left(\sum_{i=1}^N\sum_{t=1}^T{X}_{it}{\underset{\_}{y}}_{it}^{+}-{\gamma}_{12}^{+\prime}\right)$$

where *X*_*it*_ and *Y*_*it*_ stand for the cointegrated variables. ${\underset{\_}{y}}_{it}^{+}$ are the modification of regressand and the corrected serial correlation terms ($\left(i.\mathrm{e}{\underset{\_}{y}}_{it}^{+}=\left({y}_{it}-{y}_i\right)-{\hat{w}}_{12}{\omega}_{22}^{-1}{\nabla}_{22}\right),$ where *ω* and ∇ stand for the estimates of long-run covariances; ${\hat{w}}_{12}$ are the long-run standard errors of the conditional process; ${\gamma}_{12}^{+\prime }={r}_{12}-{\hat{w}}_{12}{\omega}_{22}^{-1}{\nabla}_{22}\Big)$.

The PMG estimation legitimates the estimation of long- and short-run effects. This estimation was presented by Pesaran et al. (1999), depicting the general specification as (Eq. 4)

4 $$L{(ECFP)}_{it}=\sum\limits_{j=1}^p\theta_{ij}L{(ECFP)}_{it-j}+\sum\limits_{j=1}^q\rho_{ij}x_{it-j}+\mu_{i}+\mu_{it}$$

Here, *x*_*it*_ are covariates *L*(*GREC*), *L*(*GREC*^2^), *L*(*PEC*), *L*(*ECI*), and *L*(*OTT*) *ρ*_*ij*_ represent the vector coefficients; *θ*_*ij*_ indicates coefficient with lags of the responding variables; *μ*_*i*_ and *u*_*it*_ are the individual effect and the error term, respectively. To investigate short-run coefficients, Pesaran et al. (1999) adopt the ARDL model equation as follows (Eq. 5):

5 $${\displaystyle \begin{array}{l}\Delta L{(ECFP)}_{it}={\alpha}_0+\sum\limits_{i=1}^p{\theta}_i\Delta L{(ECFP)}_{t-i}+\sum\limits_{i=1}^p{\delta}_i\Delta L{(GREC)}_{t-j}+\sum\limits_{m=1}^p{\beta}_m\Delta L{\left({GREC}^2\right)}_{t-m}\\\sum\limits_{r=1}^q{\varphi}_i\Delta L{(PEC)}_{t-r}+\sum\limits_{s=1}^p{\sigma}_i\Delta L{(ECI)}_{t-s}+\sum\limits_{z=1}^p{\sigma}_m\Delta L{(OTT)}_{t-z}+{\tau ECT}_{t-1}+{\varepsilon}_{it}\end{array}}$$

with *p* as lag of the outcome variable, *q* is the lag of the predictor, and *ECT* represents the error-correction term. Pesaran et al. (1999) suggests that PMG estimator involves averaging and pooling and acts as an intermediate estimator between mean group (MG) estimator and dynamic fixed effect (DFE) estimator. In addition, PMG estimator is evidenced to be more efficient than MG estimator under the long-run homogeneity (Pesaran et al. 1999). To decide between PMG and DFE estimator, this study relied on Hausman test. As suggested by Rahman et al. (2021), PMG, MG, and DFE estimators may produce biased results in the case of serial correlation and endogeneity issues. To overcome these problems and provide efficient estimations, our study proposes the FMOLS.

Considering the last step, we use the panel vector error correction model (VECM) framework to infer the short- and long-term causalities. The VECM model can be specified as (Eqs. 6–11)

6 $$\begin{array}{l}\Delta\mathrm L{(ECFP)}_{it}=\vartheta_{1i}+\sum\limits_m\vartheta_{11im}\Delta L{(ECFP)}_{it-m}+\sum\limits_m\vartheta_{12im}\Delta L{(GREC)}_{it-m}\\\;\;\;\;\;\;\;\;\;\;\;\;\;\;\;\;\;\;\;\;\;\;\;\;\;+\sum\limits_m\vartheta_{13im}\Delta L{\left({GREC}^2\right)}_{it-m}+\sum\limits_m\vartheta_{14im}\Delta L{(PEC)}_{it-m}\\\;\;\;\;\;\;\;\;\;\;\;\;\;\;\;\;\;\;\;\;\;\;\;\;\;+\sum\limits_m\vartheta_{15im}\Delta L{(ECI)}_{it-m}+\sum\limits_m\vartheta_{16im}\Delta L{(OTT)}_{it-m}+\tau_{1i}{ECT}_{it-1}+\varepsilon_{1it}\end{array}$$

7 $${\displaystyle \begin{array}{l}\Delta \mathrm{L}{(GREC)}_{it}={\vartheta}_{2i}+\sum\limits_m{\vartheta}_{21 im}\Delta L{(ECFP)}_{it-m}+\sum\limits_m{\vartheta}_{22 im}\Delta L{(GREC)}_{it-m}\\ {}\kern7em +\sum\limits_m{\vartheta}_{23 im}\Delta L{\left({GREC}^2\right)}_{it-m}+\sum\limits_m{\vartheta}_{24 im}\Delta L{(PEC)}_{it-m}\\ {}\kern7em +\sum\limits_m{\vartheta}_{25 im}\Delta L{(ECI)}_{it-m}+\sum\limits_m{\vartheta}_{26 im}\Delta L{(OTT)}_{it-m}+{\tau}_{2i}{ECT}_{it-1}+{\varepsilon}_{2 it}\end{array}}$$

8 $${\displaystyle \begin{array}{l}\Delta \mathrm{L}{\left({GREC}^2\right)}_{it}={\vartheta}_{3i}+\sum\limits_m{\vartheta}_{31 im}\Delta L{(ECFP)}_{it-m}+\sum\limits_m{\vartheta}_{32 im}\Delta L{(GREC)}_{it-m}\\ {}\kern6.5em +\sum\limits_m{\vartheta}_{33 im}\Delta L{\left({GREC}^2\right)}_{it-m}+\sum\limits_m{\vartheta}_{34 im}\Delta L{(PEC)}_{it-m}\\ {}\kern6.5em +\sum\limits_m{\vartheta}_{35 im}\Delta L{(ECI)}_{it-m}+\sum\limits_m{\vartheta}_{36 im}\Delta L{(OTT)}_{it-m}+{\tau}_{3i}{ECT}_{it-1}+{\varepsilon}_{3 it}\end{array}}$$

9 $${\displaystyle \begin{array}{l}\Delta \mathrm{L}{(PEC)}_{it}={\vartheta}_{4i}+\sum\limits_m{\vartheta}_{41 im}\Delta L{(ECFP)}_{it-m}+\sum\limits_m{\vartheta}_{42 im}\Delta L{(GREC)}_{it-m}\\ {}\kern6.25em +\sum\limits_m{\vartheta}_{43 im}\Delta L{\left({GREC}^2\right)}_{it-m}+\sum\limits_m{\vartheta}_{44 im}\Delta L{(PEC)}_{it-m}\\ {}\kern6.25em +\sum\limits_m{\vartheta}_{45 im}\Delta L{(ECI)}_{it-m}+\sum\limits_m{\vartheta}_{46 im}\Delta L{(OTT)}_{it-m}+{\tau}_{4i}{ECT}_{it-1}+{\varepsilon}_{4 it}\end{array}}$$

10 $${\displaystyle \begin{array}{l}\Delta \mathrm{L}{(ECI)}_{it}={\vartheta}_{5i}+\sum\limits_m{\vartheta}_{51 im}\Delta L{(ECFP)}_{it-m}+\sum\limits_m{\vartheta}_{52 im}\Delta L{(PEC)}_{it-m}\\ {}\kern6.5em +\sum\limits_m{\vartheta}_{53 im}\Delta L{\left({GREC}^2\right)}_{it-m}+\sum\limits_m{\vartheta}_{54 im}\Delta L{(PEC)}_{it-m}\\ {}\kern6.5em +\sum\limits_m{\vartheta}_{55 im}\Delta L{(ECI)}_{it-m}+\sum\limits_m{\vartheta}_{56 im}\Delta L{(OTT)}_{it-m}+{\tau}_{5i}{ECT}_{it-1}+{\varepsilon}_{5 it}\end{array}}$$

11 $${\displaystyle \begin{array}{l}\Delta \mathrm{L}{(OTT)}_{it}={\vartheta}_{6i}+\sum\limits\limits_m{\vartheta}_{61 im}\Delta L{(ECFP)}_{it-m}+\sum\limits\limits_m{\vartheta}_{62 im}\Delta L{(GREC)}_{it-m}\\ {}\kern6.75em +\sum\limits\limits_m{\vartheta}_{63 im}\Delta L{\left({GREC}^2\right)}_{it-m}+\sum\limits\limits_m{\vartheta}_{64 im}\Delta L{(PEC)}_{it-m}\\ {}\kern6.75em +\sum\limits\limits_m{\vartheta}_{65 im}\Delta L{(ECI)}_{it-m}+\sum\limits\limits_m{\vartheta}_{66 im}\Delta L{(OTT)}_{it-m}+{\tau}_{6i}{ECT}_{it-1}+{\varepsilon}_{6 it}\end{array}}$$

where Δ and *m* depict the first difference and the lag’s length, respectively.

## Results and discussion

Given the socio-economic similarities among sampled top 10 most complex economies around the globe, it is not surprising that these countries may show cross-sectional dependence. Thus, our study utilizes the cross-section dependence test proposed by Pesaran (2004) and outlines the outcome in Table 3.

**Table 3:** Pesaran (2004) cross-section dependence test

Test stat/Var./Model	L(ECFP)	L(GREC)	L(GREC^2^)	L(PEC)	L(ECI)	L(OTT)	Model
Pesaran CD	7.30^***^	28.43^***^	28.42^***^	11.51^***^	17.21^***^	21.46^***^	9.98^***^

Source: authors’ compilation from Global Footprint Network (2021), Simoes and Hidalgo (2021), World Bank (2021), and IEA (2021)

***, **, and * represent statistical significance at the 1%, 5%, and 10%, respectively

The findings, as portrayed in Table 3, assert the existence of CD which indicates the rejection of null hypothesis at 0.01 level. This implies using CIPS to test for the unit root. The results are reported in Table 4.

**Table 4:** Results of the CIPS test

Var.	Level		First difference	
	Without trend	Trend	Without trend	Trend
L(ECFP)	−3.35^***^	−3.83^***^	−5.18^***^	−5.21^***^
L(GREC)	−1.64	−2.35	−3.11^***^	−3.12^**^
L(GREC^2^)	−1.61	−2.32	−3.11^***^	−3.12^**^
L(PEC)	−1.36	−2.39	−4.27^***^	−4.56^***^
L(ECI)	−3.11^***^	−2.89^**^	−4.13^***^	−4.49^***^
L(OTT)	−1.77	−2.21	−3.35^***^	−3.29^***^

Source: authors’ compilation from Global Footprint Network (2021), Simoes and Hidalgo (2021), World Bank (2021), and IEA (2021)

***, **, and * represent statistical significance at the 1%, 5%, and 10%, respectively

Considering the cross-sectional dependence, the panel unit root CIPS test seems to yield reliable outcomes. The estimates from Table 4 affirm that all the variables are non-stationary at levels. The CIPS test, however, suggests that variables are stationary at first differences in both cases (without trend, trend). However, ecological footprint and economic complexity index are stationary at levels and first differenced in both cases (without trend, trend). Hence, the variables are I (1) as confirmed by the CIPS

After this, the cointegration test of Westerlund (2007) and Kao (1999) residual cointegration tests are adopted to affirm the cointegrating linkage between the inspected variables. It is noteworthy that Westerlund (2007) cointegration test is robust in taking CD into account. Table 5 reports the outcomes.

**Table 5:** Panel cointegration tests

Test	Statistic	Value
Westerlund (2007)	Gt	−4.529^***^
	Ga	−6.018
	Pt	−12.038^***^
	Pa	−6.479
Kao (1999)	t-stat	−3.054^***^

Source: authors’ compilation from Global Footprint Network (2021), Simoes and Hidalgo (2021), World Bank (2021), and IEA (2021)

***, **, and * represent statistical significance at the 1%, 5%, and 10%, respectively

The outcome of the Westerlund (2007) panel cointegration test with ecological footprint being the dependent variable suggests a long-run association for the variables analyzed in this study. Concerning the *p* values, the null hypothesis is rejected to authenticate the long-run relationships between the variables for the stats Gt and Pt at the 1% statistical significance, while the null hypothesis for stats Ga and Pa is upheld. The outcome with that of Kao (1999) residual cointegration test authenticates Westerlund’ result for panel cointegration test and confirms the presence of cointegration among the analyzed factors for the top 10 most complex economies around the globe. This indicates that the suggested predictor varies long-run relationship with ecological impediments. These findings served to answer our research question 2.

Since cointegration relationship was affirmed amid variables concerning the Westerlund (2007) panel cointegration test and Kao (1999) residual cointegration test, we use two different techniques such as the PMG and FMOLS to estimate empirically the corresponding parameters of the predictor variables. Table 6 presents the estimation results.

**Table 6:** Results of FMOLS and PMG-ARDL estimation

Variables/estimation method (long-run equation)	FMOLS	PMG (*p* = *q* = 1)
	Pooled	
L(GREC)	4.738^***^ (1.269)	8.075^***^ (2.512)
L(GREC^2^)	−0.220^***^ (0.059)	−0.337^***^ (0.123)
L(PEC)	0.607^***^ (0.100)	0.712^***^ (0.128)
L(ECI)	−0.423^***^ (0.080)	−0.237^***^ (0.070)
L(OTT)	−0.162^***^ (0.044)	−0.370^***^ (0.063)
Variables/estimation method (short-run equation)		PMG (*p* = *q* = 1)
ECT_−1_		−0.736^***^ (0.141)
ΔL(GREC)		−12.908 (30.644)
ΔL(GREC^2^)		0.481 (1.446)
ΔL(PEC)		−0.690^**^ (0.318)
ΔL(ECI)		−0.148 (0.122)
ΔL(OTT)		0.311^***^ (0.111)
Constant		−36.885^***^ (7.062)
Trend		−0.011^***^ (0.003)
Groups	10	10
Observations	190	190
R-squared	0.877	
Sum-squared resid	0.757	0.231
Log-likelihood		447.518

Source: authors’ compilation from Global Footprint Network (2021), Simoes and Hidalgo (2021), and World Bank (2021), IEA (2021)

***, **, and * represent statistical significance at the 1%, 5%, and 10%, respectively

Particularly, the empirical evidence of the FMOLS portrays that real GDP per capita and ecological footprint are positively and significantly related where the square of real GDP per has a reverse association with the ecological footprint. Hence, we can certify the authenticity of the EKC hypothesis for the top 10 most complex economies around the globe. The results show that higher real GDP per capita boosts the ecological impediment. Besides, at a certain level, increased real GDP per capita curbs ecological impediment. These findings served to answer our research question 1. On the other side, the coefficient of electric power consumption is positive and significant. Thus, a percentage increase in electric power consumption triggers the ecological footprint to increase by 0.607%. In addition, both economic complexity index and trade openness are evidenced to have a negative coefficient.

The PMG-ARDL estimation is further employed to investigate the robustness of the FMOLS estimations. Table 6 outlines the outcome of PMG-ARDL estimation. The results from the two estimations (FMOLS and PMG-ARDL) are in harmony. They confirm that *GREC* adds to ecological impediments in the inspected countries. The parameter estimates of *GREC*^2^, in addition, are negative, thus inferring that the prediction of the EKC hypothesis holds, i.e., inverted U-shape relationship is persistent in the long run. Economic implications of these findings show that in the early phases of economic growth in the top 10 most complex economies around the globe, special attention has been given to the economic expansion, whereas environmental challenges are ignored. However, this liaison is broken at a certain level, meaning that real GDP per capita reduces ecological impediment. Our results affirm the authenticity of studies by Muslija et al. (2020), Chu (2020), Adams et al. (2020), Satrovic et al. (2021), Mahmoodi and Dahmardeh (2022), and Qamar et al. (2022). However, these findings are in contrast with Bese and Friday (2022) demonstrating no inverted U relationship between ecological footprint and external debt.

Undoubtedly, the empirical proof further shows that the electric power consumption in the inspected complex economies would lead to the increase in ecological footprint by 0.607% (FMOLS) and 0.712% (PMG-ARDL) at a 1% connotation level, ceteris paribus. Like real GDP per capita, electric power consumption is one of the key predictors of environmental degradation. The expansion of the economy increases electric power consumption, deteriorating the environment which is closely linked with the anthropogenic emissions. This finding aligns with Khan et al. (2019), Neagu (2020), Satrovic et al. (2020), Mahmoodi and Dahmardeh (2022), and Massagony and Budiono. (2022). Conversely, the economic complexity index is evidenced to have a negatively significant effect on ecological footprint. Statistically, ceteris paribus, a percentage increase in economic complexity index enhancement reduces ecological footprint in the inspected countries by 0.423% (FMOLS) and 0.237% (PMG-ARDL). We found beneficial environmental effect of economic complexity in the selected countries. Our results support the view that the improved productive sophistication of a country increases investments in cleaner technology and positively impacts environmental quality. The empirical evidence on the effects of ECI on environmental degradation is like the previous findings of Doğan et al. (2019), Laverde-Rojas et al. (2021), and Wan et al. (2022).

For trade openness, there is a linear liaison such that ecological footprint decreases significantly by −0.162% (FMOLS) and −0.370% (PMG-ARDL) for every 1% increase in trade openness in the long run, ceteris paribus. There is no doubt that these findings support the view that trade is one of the drivers of environmental quality. This may happen since most complex countries improved productive sophistication and enacted good environmental initiatives to reduce the production of polluting goods. In addition, this may happen since developed countries begin to relocate their hazardous and polluting industries to developing economies. The finding of negative coefficients for trade openness is in line with the findings of Doğan et al. (2019), Tachie et al. (2020), Laverde-Rojas et al. (2021), Boleti et al. (2021), and Andriamahery and Qamruzzaman (2022).

The PMG-ARDL estimate makes it possible to derive both long-run and short-run causality conclusions. The error correction term has a statistically significant negative value of 1%. This result implies that in the event of a systemic change or shocks, about 73.6% of the disequilibrium converges to the long-run equilibrium. Keeping other variables constant, a 1% increase in economic growth, its square, and economic complexity index do not affect the environment in the short run. Unlike these findings, electric power consumption is one of the drivers of environmental quality, whereas trade openness drives, in the short run, the environmental dilapidation. The Hausman test results suggest no rejection of null hypothesis and recommends PMG over MG estimator. In contrast, the findings suggest that MG should be selected over DFE. Given that the Hausman test between DFE and PMG fails to meet the asymptotic assumptions of the Hausman test, the PMG is selected as the more efficient for our analysis and we report these findings accordingly.

Moreover, we use the panel VECM framework to study the pattern of causal connection between ecological footprint and ECI as well as other predictors. Table 7 displays the results.

**Table 7:** Summary results of VECM (*m* = 2)

Dependent variables	Short-run coefficients						Long-run coefficients
	Δ*L*(ECFP)	Δ*L*(GREC)	Δ*L*(GREC^2^)	Δ*L*(PEC)	Δ*L*(ECI)	Δ*L*(OTT)	ECT
Δ*L*(ECFP)	-	−0.070^**^	−1.427^**^	−0.077^***^	0.344^***^	−0.147^*^	−1.858^***^
		(−0.028)	(−0.580)	(−0.029)	(−0.082)	(−0.079)	[−9.206]
Δ*L*(GREC)	−8.857^**^	-	−17.552	1.872	−3.023	−3.831	−0.054^***^
	(−4.005)		(−31.563)	(−1.493)	(−2.315)	(−4.412)	[−10.438]
Δ*L*(GREC^2^)	−0.427^**^	0.050	-	−0.087	0.139	0.370^*^	−0.029^***^
	(−0.191)	(−0.073)		(−0.071)	(−0.110)	(−0.213)	[−5.988]
Δ*L*(PEC)	−0.339	0.197^*^	3.949^*^	-	−0.037	0.811^**^	−0.535^***^
	(−0.290)	(−0.111)	(−2.289)		(−0.168)	(−0.325)	[−4.437]
Δ*L*(ECI)	0.582^***^	−0.242^***^	−5.108^***^	−0.211^***^	-	−0.182^*^	−0.443^***^
	(−0.149)	(−0.057)	(−1.177)	(−0.056)		(−0.109)	[−3.802]
Δ*L*(OTT)	0.501^***^	0.059	1.234	0.081^**^	−0.025	-	−0.259^***^
	(−0.098)	(−0.037)	(−0.769)	(−0.036)	(−0.056)		[-5.110]
Observations	160						
R-squared	0.800	0.234	0.482	0.284	0.390	0.539	---
S.E. of regression	0.067	0.025	0.529	0.025	0.035	0.075	---
F-statistic	8.862	10.119	10.456	5.189	5.756	2.379	---
Log likelihood	228.666	366.699	−117.765	390.861	300.266	218.566	---

Source: authors’ compilation from Global Footprint Network (2021), Simoes and Hidalgo (2021), World Bank (2021), and IEA (2021)

***, **, and * represent statistical significance at the 1%, 5%, and 10%, respectively

Table 7 summarizes the causal relationships between the inspected variables. Particularly, results presented in Table 7 suggest a bidirectional causal relationship between (1) *GREC* and ecological footprint; (2) *GREC*^2^ and ecological footprint; (3) economic complexity index and ecological footprint; and (4) trade openness and ecological footprint. However, the findings evidenced a one-way causal linkage running from electric power consumption to ecological footprint. These findings are consistent with Adedoyin and Zakari (2020) in the top ten international tourist earners; Tachie et al. (2020) in developed nations; and Laverde-Rojas et al. (2021) in Colombia.

## Conclusion and policy recommendation

In the last few decades, complex economies have boosted their complexity to higher levels. More complex and sophisticated exported products are a demand to face the challenge in a world of increasingly global competition. The higher complexity of products may rapidly increase global energy demand and causes environmental challenges. In this section, in addition to discussing the economic impacts of the economic complexity index, there are also environmental effects as well. On this basis, our study uses annual panel data for the top 10 most complex economies around the globe from 1998 to 2017 to investigate the impact of electric power consumption and economic complexity index while accounting for other macroeconomic indicators of ecological footprint. To that purpose, our article explores the EKC hypothesis’s validity.

The EKC hypothesis was found to be valid in a panel of the world’s ten most sophisticated economies according to empirical findings. The findings of this paper support the argument that in the long run, electric power consumption further reduces the quality of our environment. In contrast, the economic complexity index and trade openness improve environmental quality. According to our findings, a country’s ecological constraints are substantially connected with the mix of its exported products. Using FMOLS and PMG-ARDL calculations, we confirmed the favorable influence of the economic complexity index on environmental quality. To this end, an increase in the “sophistication” of a country’s production and of its exports is positively associated with environmental quality. We also found bidirectional causality between (1) economic growth and capita and ecological footprint; (2) the square of economic growth and ecological footprint; (3) economic complexity index and ecological footprint; and (4) trade openness and ecological footprint, and one-way causality flowing from electric power consumption to ecological footprint.

The current study suffers from some drawbacks; some of them can be addressed in future studies. Although every effort was made to gather as much data as possible, the current study is constrained in that it planned to use a timespan that was greater than what was used, but owing to data restrictions for some variables, the investigated timespan was limited to 1998 to 2017. Furthermore, this study is constrained in that it was conducted on a panel case without considering the situation of a single nation. Finally, future studies on the implications of economic complexity on environmental footprint should investigate the function of economic policy uncertainty in different nation samples. Future studies may consider stochastic effect by inference on population, affluence, and technology (STIRPAT) framework, as the foundation of their analysis. Considering the advantages of panel quantile regression over ordinary least squares, it is highly recommended to be employed in future studies investigating the determinants of environmental degradation.

Based on these findings, a few policy implications can be deduced. It is pertinent that policymakers should formulate policies that prohibit non-renewable energy sources and support the usage of renewable energy that will have positive environmental effects. More specifically, policymakers should concentrate on long-term green regulation instead of setting superfluous objectives in response to disruptions in the carbon footprint. From a policy aspect, electricity consumption is indeed an important variable that policy should watch for in maintaining a sustainable environment. To that aim, expenditures in technical advances should be enhanced since renewable energy sources are critical to ensuring long-term energy security. Furthermore, economic complexity improves environmental quality. It is therefore reasonable for the inspected top 10 complex countries around the globe to promote the “sophistication” of a country’s production that will encourage investments in energy-efficient technologies. Given the negative liaison between trade openness and ecological footprint, policymakers need to put in place environmental provisions in their trade agreements to reduce environmental challenges. Technological advancements must be complemented by legislative standards that govern pollution emissions. Given that economic complexity can improve environmental sustainability when economic openness is in place in the most complex countries, it is recommended to integrate economic openness policies and sophistication of products with the environmental policy framework.

## Data Availability

The datasets generated and/or analyzed during the current study are not publicly available due but are available from the corresponding author on reasonable request.
